# An evaluation of common methods for comparing the scaling of vertical force production in flying insects

**DOI:** 10.1016/j.cris.2022.100042

**Published:** 2022-07-13

**Authors:** Nicholas P. Burnett, Emily L. Keliher, Stacey A. Combes

**Affiliations:** Department of Neurobiology, Physiology, and Behavior, University of California, Davis, Davis, CA, 95616

## Abstract

Maximum vertical force production (F_vert_) is an integral measure of flight performance that generally scales with size. Numerous methods of measuring F_vert_ and body size are accessible to entomologists, but we do not know whether method selection affects inter- and intraspecific comparisons of F_vert_-size scaling. We compared two common techniques for measuring F_vert_ in bumblebees (*Bombus impatiens*) and mason bees (*Osmia lignaria*), and examined F_vert_ scaling using five size metrics. F_vert_ results were similar with incremental or asymptotic load-lifting, but scaling analyses were sensitive to the size metric used. Analyses based on some size metrics indicated similar scaling exponents and coefficients between species, whereas other metrics indicated coefficients that differed by up to 18%. Furthermore, F_vert_ showed isometry with body lengths and fed and starved masses, but negative allometry with dry mass. We conclude that F_vert_ can be measured using either incremental or asymptotic loading but choosing a size metric for scaling studies requires careful consideration.

## Highlights

• Two methods to measure max vertical force production give similar values in bees
• Interspecific differences in force-scaling depend on the body size metric used
• Only dry mass shows interspecific differences in relative force (% of mass lifted)

## Introduction

Maximum vertical force production (F_vert_) is an integral component of flight performance, and has been examined across a diversity of volant taxa (Marden, 1987). To maintain flight altitude, animals must produce vertical forces that at least match their body weight (mass*gravitational acceleration), and elaborate flight behaviors require additional force production_._ For instance, animals that produce vertical forces exceeding their body weight can engage in vertical acceleration (e.g., evasive flight maneuvers) or load-carrying (e.g., transporting food or nesting materials) (Buchwald and Dudley, 2010; Marden, 1987; Wolf and Schmid-Hempel, 1989). Across many birds, bats, and insects, F_vert_ scales isometrically with flight muscle mass, which generally increases with body size (Marden, 1987), but inter- and intraspecific variation in F_vert_ scaling can exist due to differences in musculature, morphology, or kinematics (Chai et al., 1997; Dillon and Dudley, 2004). Although previous studies have compared techniques for measuring F_vert_ or size in scaling analysis (Buchwald and Dudley, 2010; Cane, 1987), these assessments explored only one of the variables (either F_vert_ or size). Thus, it is unknown how the different methodologies that are common to insect flight studies influence the outcomes of studies comparing F_vert_ scaling.

### Measuring vertical force production

The two simplest methods of measuring F_vert_, incremental and asymptotic load-lifting, involve challenging animals to sustain flight with the heaviest added load possible. In the incremental method, weights are attached to an animal and the animal is prompted to fly. After each successful flight, additional weights are added. This process is repeated until the animal can no longer fly, and the maximum load (body + added weights) reached before failure defines F_vert_. The incremental method has been used on bats, birds, and insects (Marden, 1987), and the bumblebee *Bombus impatiens* (Buchwald and Dudley, 2010). In the asymptotic method, a beaded string (small masses attached to a string at fixed intervals) is attached to an animal and the animal is prompted to fly vertically. As the animal takes off and increases altitude, it lifts more of the beaded string until it is unable to lift additional mass; the weight of the animal's body plus the beads and string lifted indicates F_vert_. This method has been used on hummingbirds (Altshuler and Dudley, 2003; Chai et al., 1997), orchid bees (Dillon and Dudley, 2004), and *B. impatiens* (Buchwald and Dudley, 2010; Mountcastle and Combes, 2013).

The asymptotic method is advantageous because F_vert_ is measured in a single flight trial, whereas the incremental method requires numerous flights (which can be time-consuming and exhaust the animal's energy reserves). However, the asymptotic method is problematic for species with erratic, non-vertical flight behaviors (Su et al., 2020). Both methods are difficult in species that cannot be handled or have a mass attached to their body (Altshuler and Dudley, 2003). Comparisons of these methods have suggested that the incremental method underestimates F_vert_ (Buchwald and Dudley, 2010), but this assessment has not been replicated or tested in additional species. Assessing the cross-compatibility of these widespread methods of measuring F_vert_ is necessary to facilitate comparative studies of species exhibiting flight behaviors that may preclude one of the methods.

### Scaling performance by size

Flying animals must produce, at minimum, enough force to support their own body weight, so F_vert_ generally increases with body size. F_vert_ often increases isometrically with body size (Buchwald and Dudley, 2010; Dillon and Dudley, 2004; Marden, 1987; Marden, 1990). In entomological research, body size is commonly quantified using a length measure (e.g., wing length, intertegular (IT) span) or a mass measure (e.g., fed, starved, or dry body mass). Are these traditional metrics interchangeable in scaling analyses of flight performance? In bees (Apoidea), IT span (distance between tegulae at the wing bases) and wing length are morphological features that are measured directly with calipers or through photographs, and are proportional to body mass in closely related taxa (Cane, 1987; Dillon and Dudley, 2004). Fed mass is the body mass measured before or after a flight trial; this measure introduces variability if insects carry different volumes of energy reserves when selected for a flight trial (Marden, 1987), which may also alter the underlying flight muscle physiology (Marden et al., 2008). Starved (or empty) mass is the insect mass without any stored energy reserves, and thus, represents the baseline body mass that must be lifted to fly; in bees, this is obtained by measuring body mass after squeezing a bee to cause regurgitation of nectar from its honey sac, or crop (Buchwald and Dudley, 2010). However, this technique introduces error because not all nectar is stored in the crop: up to 10% is retained in the midgut after regurgitation (Gary and Lorenzen, 1976). Alternatively, empty mass is obtained by weighing insects after starving over some time period (e.g., 24 hours) to allow all energy reserves in the body to be metabolized while avoiding desiccation or mortality (Combes et al., 2020). Dry mass is the body mass after desiccating a dead insect to a constant mass in an oven (Cane, 1987; Helm et al., 2021); this method introduces error because energy reserves (or other materials) may remain in the insect after desiccation (especially if the insect was not starved beforehand), adding to the dry mass. Although IT span, wing length, and fed, starved, and dry masses are among the simplest and most widespread body size measurements used in insect flight studies, the variability introduced by each of these metrics has not been compared across species in the context of flight performance.

### Study system

We compare two simple methodologies for quantifying F_vert_, by performing both measurements on females of two bee species, the eastern bumblebee *Bombus impatiens* and the mason bee *Osmia lignaria*. We test whether interspecific comparisons of flight performance, controlled for body size, depend on the size metric used in the analyses. These species are in the superfamily Apoidea but differ in body size (most *O. lignaria* females are smaller than *B. impatiens* workers), morphology, and life history (*O. lignaria* are solitary and B*. impatiens* are primitively eusocial). *Bombus impatiens* is an established model organism for flight biomechanics studies (Buchwald and Dudley, 2010; Combes et al., 2020; Mountcastle and Combes, 2013), and *O. lignaria* is an emerging model for studies of flight biomechanics, reproductive physiology, and landscape ecology (Bosch and Kemp, 2000; Helm et al., 2021; Kemp et al., 2004; Vicens and Bosch, 2000). Both species are sold commercially for use in crop pollination as an alternative to honeybees. Thus, these species are not closely related but may be inadvertently grouped together in broad analyses of flight performance across taxa.

## Materials and methods

Cocoons of adult-wintering *Osmia lignaria* were purchased from a commercial supplier (Foothill Bee Ranch, Auburn, CA, USA) and maintained at 4°C. Individuals were moved to a flight cage for emergence, as needed for experiments. A mature colony of *Bombus impatiens* was purchased from a commercial supplier (Koppert Biological Systems, Romulus, MI, USA) and maintained in a separate flight cage. Individuals in each cage were fed sucrose solution *ad libitum* with fresh pollen weekly. Flight cages and experimental areas were held at 22-25°C. Active females of each species were selected randomly for trials (n = 25 *O. lignaria,* 3-14 days post-emergence; 28 *B. impatiens*, age unknown).

### Flight performance

**Figure 1:**
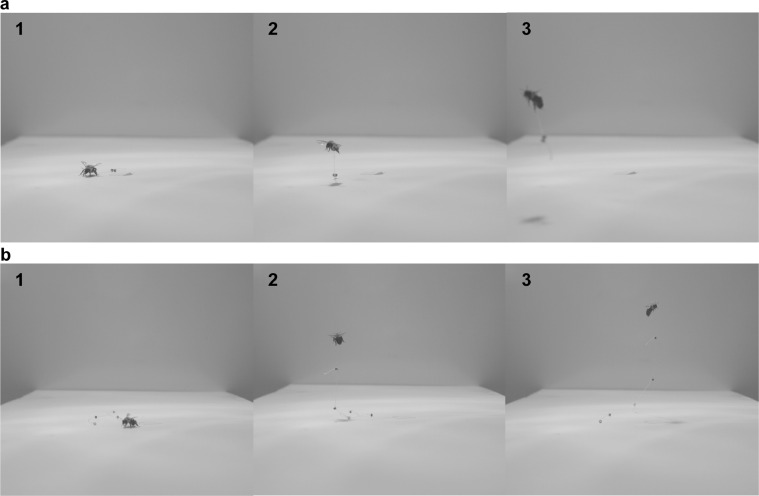
Examples of the (a) incremental and (b) asymptotic methods for measuring maximum vertical force production. Each example shows a three-photograph sequence of a single flight attempt, using a female *Bombus impatiens.*

F_vert_ was measured on each individual using both the incremental and asymptotic methods, to allow for direct comparison (Fig. 1). Order of the methods was alternated between individuals, with both tests performed during the same day. Testing methodology is briefly described in the Introduction (“*Measuring vertical force production*”) and generally followed descriptions by Buchwald and Dudley (2010) and Mountcastle and Combes (2013). Detailed protocols for each method are described in Supplementary File S1. F_vert_ for each method was calculated as the sum of the bee mass (averaged between pre- and post-flight mass) and the lifted mass of beads (incremental method) or beaded string (asymptotic method), multiplied by gravitational acceleration. We consider the maximum lifted mass to be the observed maximum lifted mass, following Mountcastle and Combes (2013). However, other studies considered maximum lifted mass to be the mean between the observed maximum lifted mass and the next-highest mass that the bee was unable to lift (Buchwald and Dudley, 2010; Marden, 1987). This variation in methodology can impact comparisons of data between studies but does not affect the conclusions of the present study because the same approach was used for all trials.

### Body size

After all flight trials using both methods were completed for each bee, the string was removed from the petiole and body mass was measured to the nearest 0.0001 g with a digital balance (providing the fed mass). The bee was placed in a separate dish with only a wet paper towel and left for 24 h at room temperature to consume any nectar remaining in its body. After 24 h, body mass was measured again (providing the starved mass), and the bee was frozen.

Once flight tests were completed, we removed bees from the freezer, photographed them, and measured their intertegular (IT) span and forewing length (hereafter wing length) to the nearest 0.01 mm using ImageJ (v1.53f51) (Schneider et al., 2012). Afterwards bees were dried to a constant mass in an oven at 45°C, providing the dry mass (Cane, 1987).

### Statistical analysis

Within each species, we compared F_vert_ measurements between the incremental and asymptotic methods using paired *t-*tests (paired by individual).

We compared F_vert_-size scaling between species using the size metrics described above. F_vert_ and size data for each species can be represented by the power function *Y* = β*X*^α^, where *Y* is F_vert_, *β* is a scaling coefficient, *X* is size, and *α* is a scaling exponent. This function can also be expressed in logarithmic form: log_10_*Y* = log_10_*β* + *α*log_10_*X*. Here, β and α are the *Y*-intercept and slope of the log_10_-transformed model, respectively (Vogel, 2013).

We log_10_-transformed data to conduct an ANCOVA (analysis of covariance) scaling analysis for each size metric, using F_vert_ as the dependent variable, species as the independent variable, and body size as the covariate. We first tested for a statistical interaction between species and size (i.e., different scaling exponents between species); if none was found, we tested for a statistical effect of species across size (i.e., different scaling coefficients between species). Force production in flight muscle scales isometrically with mass, rather than mass^2/3^ (with muscle cross-sectional area) as predicted for isometric muscle contraction (Marden, 1987; Schilder and Marden, 2004). Thus, for each size metric we compared F_vert_ scaling exponents to predictions of 1 (for body masses) and 3 (for body lengths) using Wald tests with the ‘linearHypothesis’ function in the R package *car*. All analyses were done in R Statistical Software (R Core Team, 2020).

## Results and Discussion

**Figure 2:**
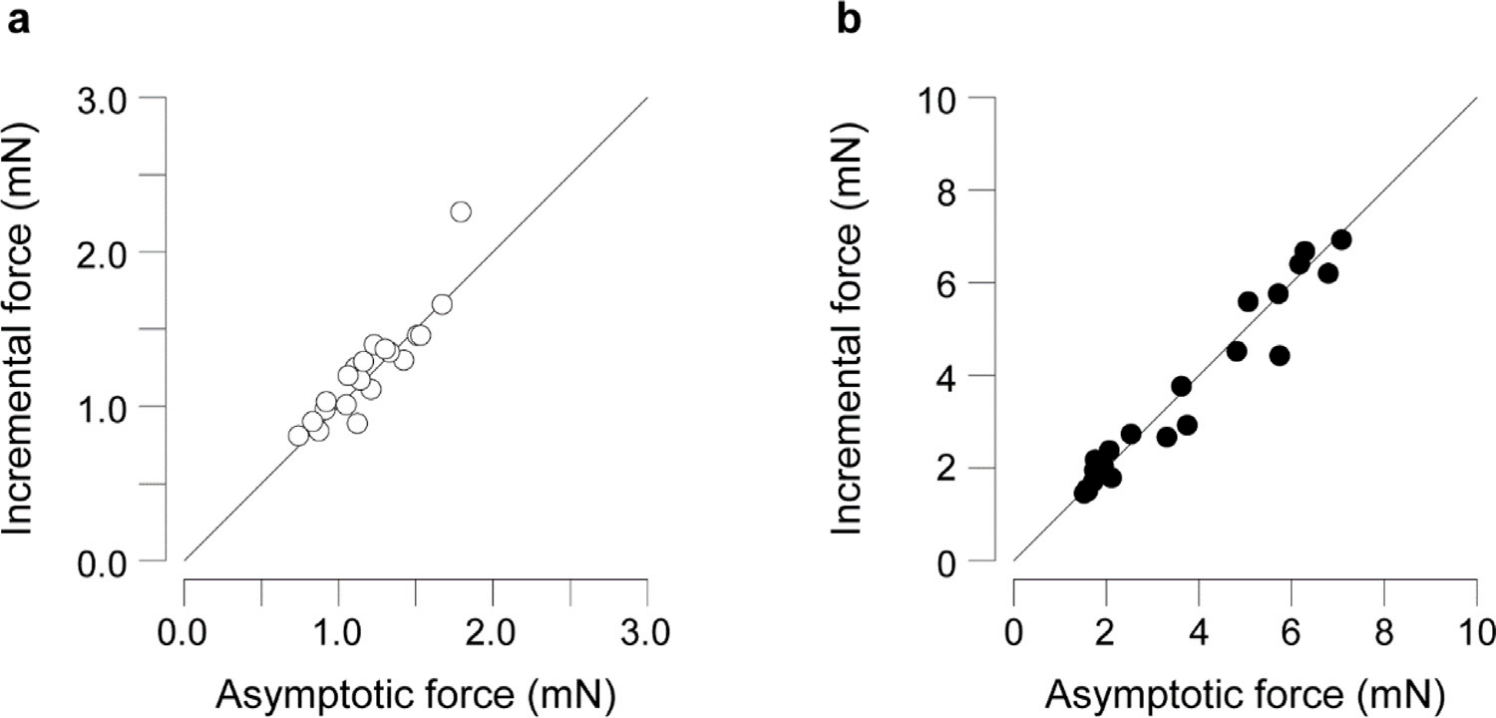
Paired measurements of vertical force production show that the incremental and asymptotic methods produce similar results. Paired F_vert_ measurements using both methods in each individual are shown for (a) *Osmia lignaria* (n = 25) and (b) *Bombus impatiens* (n = 28). Horizontal axes show F_vert_ measured with the asymptotic method and vertical axes show F_vert_ measured with the incremental method. The line in each panel shows a slope = 1. In both cases, incremental and asymptotic methods produced statistically similar results (paired *t-*tests, *p* > 0.05).

F_vert_ scaling depended on the size metric used but not on the F_vert_ method used. The incremental and asymptotic methods produced similar results within each species (Fig. 2). The methods differed by 3.0±10.4% (mean±SD) for *Osmia lignaria*, calculated as asymptotic – incremental, divided by the mean of the two methods (paired *t-*test, *p* = 0.206), and 1.6±12.6% for *Bombus impatiens* (*p* = 0.428).

There were no differences in scaling exponent *α* (i.e., slope of log_10_-transformed data) between species for any size metric (ANCOVA, *p* > 0.05). However, scaling coefficient *β* (i.e., intercept of log_10_-transformed data) differed significantly between species (*p* < 0.005) when IT span or dry mass was the size metric. *β* was similar between species with the other size metrics.

**Figure 3:**
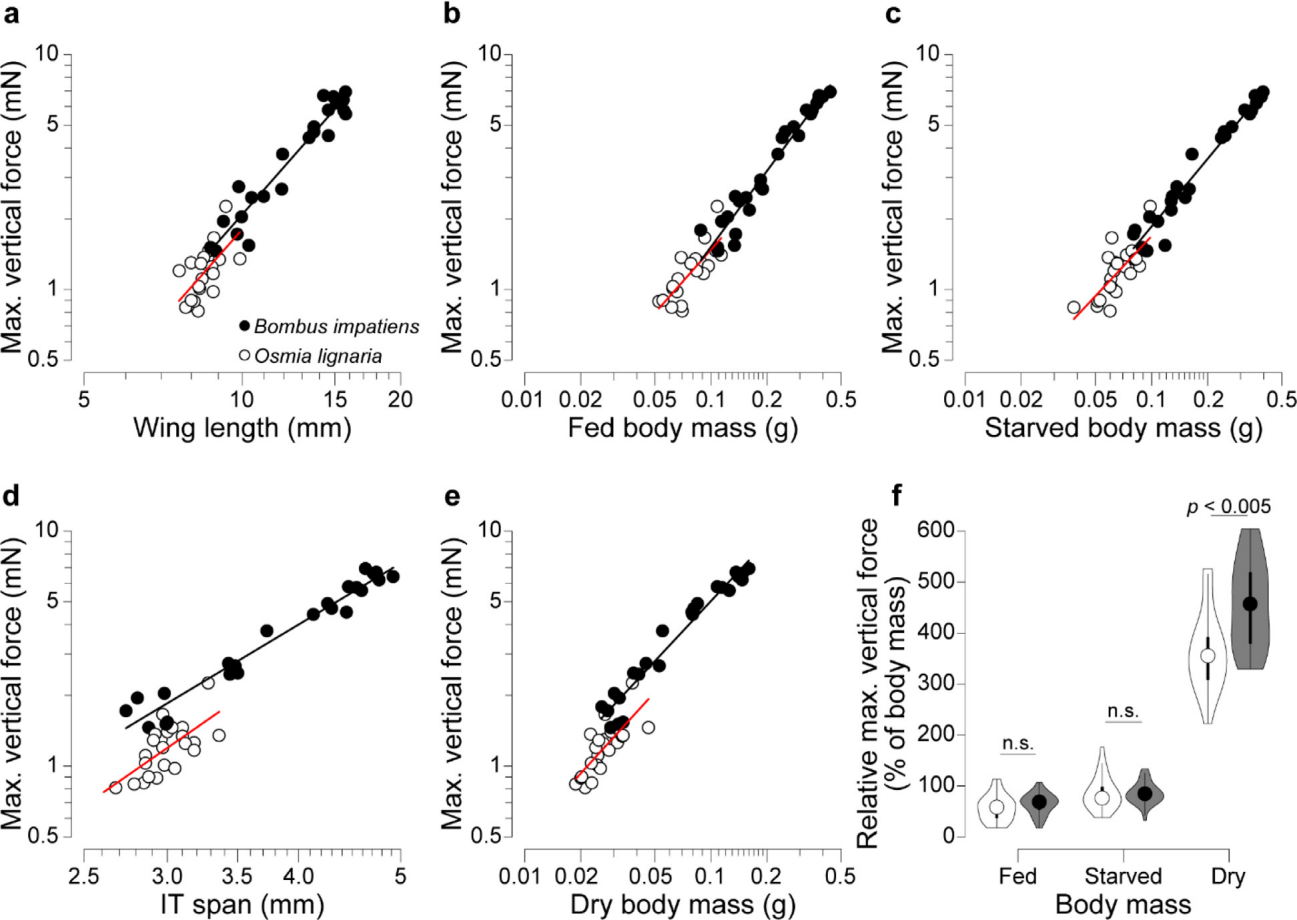
Inter-specific scaling analyses of maximum vertical force production lead to different conclusions depending on the size metric used. *Bombus impatiens* and *Osmia lignaria* display statistically similar scaling exponents and coefficients when F_vert_ is expressed as a function of (a) wing length, (b) fed body mass, or (c) starved body mass (ANCOVA, *p* > 0.05). The two species display similar scaling exponents but significantly different coefficients (i.e., *Y*-intercepts) when F_vert_ is expressed as a function of (d) IT span or (e) dry body mass (*p* < 0.005). (f) Mass-specific F_vert_ is similar in *B. impatiens* and *O. lignaria* if F_vert_ is normalized to fed or starved mass, but significantly larger in *B. impatiens* if F_vert_ is normalized to dry mass (*t*-tests, *p* < 0.05 for significance). In (f), circles show medians, bars show 25^th^ and 75^th^ percentiles, and violin plots shown the kernel density-smooth representations of the frequency distributions. White symbols represent *O. lignaria*, and black/gray symbols represent *B. impatiens.*

With the incremental method (asymptotic results are similar), F_vert_ scaled isometrically (expected *α* = 3) with wing length (*α* = 2.778; *p* = 0.053) and IT span (*α* = 2.711; *p* = 0.079). F_vert_ also scaled isometrically (expected *α* = 1) with fed mass (*α* = 1.052; *p* = 0.121) and starved mass (*α* = 0.980; *p* = 0.506; Fig. 3) but showed negative allometry with dry mass (*α* = 0.851; *p* < 0.005). With IT span and dry mass, the scaling coefficient *β* was 0.187 and 0.137 lower, respectively, for *O. lignaria* than for *B. impatiens* (*p* < 0.005).

F_vert_ scaling was nearly identical between *O. lignaria* and *B. impatiens* when wing length, fed mass, and starved mass were used. Thus, analyses using these metrics suggest that *B. impatiens* produces larger F_vert_ primarily because it is larger than *O. lignaria*, and that both species have similar F_vert_ when normalized to fed or starved mass (paired *t-*tests, *p* > 0.05; Fig. 3f). However, IT span and dry mass show different scaling coefficients, and F_vert_ normalized to dry mass differs significantly between species (*p* < 0.005). So while F_vert_ scales isometrically with size in each species, interspecific variation in scaling coefficients suggests that F_vert_ is shaped by other factors, such as physiology or kinematics.

### Considerations for future studies

We show that the incremental and asymptotic methods of measuring F_vert_ produce equivalent results for *B. impatiens* and *O. lignaria*. Thus, either method accurately measures F_vert_, and researchers can choose the most feasible method given their study subjects’ flight behavior. However, other comparative studies found different patterns. For instance, Buchwald and Dudley (2010) showed that the incremental method underestimated F_vert_ in *B. impatiens*, a result possibly due to their different method of applying incremental weights (gluing versus tying weights to bees). F_vert_ measurements also depend on whether assays involve a steady flight behavior (hovering or slow, level flight) or a dynamic flight behavior (rapid accelerations). For instance, when loaded with weights and dropped, the dragonfly *Pantala flavescens* exhibits a dynamic “pull-up” behavior (rapid acceleration as it stops its descent and ascends upwards) that produces F_vert_ much larger than anything produced during sustained flight behavior (Su et al., 2020). Thus, F_vert_ measurements based on sustained hovering or steady flight may be broadly incompatible with measurements based on dynamic flight.

Size metrics may not always be interchangeable or comparable, especially between distantly related species. For instance, IT span is useful for comparing size within bee species, but tegulae (and thus IT span) are only found in certain insect groups. Single linear dimensions of animals may also be misleading and not capture three-dimensional differences in morphology between species or across ontogeny. In past studies, scaling of flight performance across large and diverse groups of organisms has used flight muscle mass (versus total body mass) because flight muscles actuate the wings (Buchwald and Dudley, 2010; Dudley, 1995; Marden, 1987; Marden, 1990). However, different species – or individuals of different sizes within a species – require different techniques for isolating flight muscle, which could bias morphological comparisons. For instance, flight muscle in bees and other insects can be quantified via dissection or chemical digestion of the thorax, and each technique has its own sources of error (e.g., correctly dissecting or digesting all of the flight muscle, and *only* flight muscle) (Buchwald and Dudley, 2010; Dudley, 1995; Marden, 1987). Thus, it is imperative for researchers to confirm that the size metrics used in inter- or intraspecific comparisons of flight performance are compatible across the range of organisms studied.

## Data Availability

Data are available from the Dryad Digital Repository: 10.25338/B8DD1Q

The R Script necessary to replicate the analyses and figures is available in the Supplementary Materials.

## CRediT authorship contribution statement

**Nicholas P. Burnett:** Conceptualization, Methodology, Investigation, Formal analysis, Data curation, Writing – original draft, Visualization. **Emily L. Keliher:** Investigation, Formal analysis, Writing – review & editing. **Stacey A. Combes:** Conceptualization, Methodology, Supervision, Project administration, Funding acquisition.

## Declaration of Competing interest

The authors declare that they have no known competing financial interests or personal relationships that could have appeared to influence the work reported in this paper.

## Appendix Supplementary materials

Image, application 1

Image, application 2
