# Supplementary material for: An evaluation of common methods for comparing the scaling of vertical force production in flying insects
Source: Curr Res Insect Sci. 2022 Jul 13;2:100042. doi: 10.1016/j.cris.2022.100042 (PMC9387496; doi:10.1016/j.cris.2022.100042)
Supplement: Supplementary file 1 [file mmc1.docx]

**File S1**

**Protocols for incremental and asymptotic measurements of maximum vertical force production in *Osmia lignaria* and *Bombus impatiens***

Part 1. Attaching a polyester thread to bee – applicable to incremental and asymptotic methods

1. Cold-anesthetize bee by placing in refrigerator at 4ºC for 10-15 minutes.
2. Cut a 20-cm length of polyester thread.
3. Once bee is fully anesthetized, remove bee from refrigerator and place on a cold pack (or any covered ice pack) to keep chilled.
4. While the bee is resting on the cold pack, use forceps to maneuver the bee and the thread. Loop the polyester thread around the petiole of the bee (i.e., the space between the abdomen and the thorax) and tie into a square knot, securing the thread around the petiole. Leave approximately 6 cm of thread hanging free on end and trimming the other end.

Part 2. Incremental method for measuring maximum vertical force production.

1. Tie individual plastic beads (either 0.0250 or 0.0050 g in mass) to the free thread hanging from the thread. Start with 1 bead on the initial trial.
2. Measure the combined mass of the bee, thread, and any attached beads.
3. Release bee into a flight arena and prompt to fly, e.g., using agitation with forceps.
4. If the bee takes off and flies for >0.5 seconds (i..e., at least ~75 wing beats for large *B. impatiens*) at a constant or increasing altitude, consider the trial a success. Return to Step 1, adding additional bead(s), and repeat the flight assay.
5. Once the bee is unable to fly with the attached mass of bees, the bee has surpassed its maximum lifting capacity.
6. Calculate maximum vertical force production as the maximum mass lifted (total of bee, thread, and beads), multiplied by gravitational acceleration.

Part 3. Asymptotic method for measuring maximum vertical force production.

1. Prepare a beaded string: Attached beads (either 0.0250 or 0.005 g in mass) to a polyester string at some fixed interval (1-3 cm). Measure the mass of the string. Calculate the mean mass per bead on the string, as total beaded string mass divided by the number of beads.
2. Measure the combined mass of the bee and its thread.
3. Attached the beaded string to the thread on the bee (e.g., via loop or square knot).
4. Release bee into a flight arena and prompt to fly, e.g., using agitation with forceps.
5. Record flights with a video camera (e.g., at 30-60 frames per second).
6. Successful trials occur when the bee lifts part of the beaded string off the ground, maintaining a constant altitude (i.e., the number of beads lifted off the ground is steady) with minimal horizontal flight (i.e., the beaded string remaining on the ground does not move) for >0.5 seconds (i.e., at least ~ 75 wing beats for large *Bombus impatiens*). Some species are more likely to hover in a stationary position than others, so the cut-offs used for each species may need to be adjusted depending on possible behaviors.
7. Obtain up to five successful flights, recorded the maximum number of beads (from the beaded string) that were lifted in each flight.
8. Remove the beaded string from the bee’s thread and measure the combined mass of the bee and its thread.
9. Calculate maximum vertical force production as the mean mass of the bee and its thread (between pre- and post-flights) plus the maximum mass of beaded string lifted (i.e., number of beads lifted multiplied by the mean mass per bead on the string), multiplied by gravitational acceleration.

**Detailed protocol for measuring body sizes of *Osmia lignaria* and *Bombus impatiens***

1. Fed body mass: Mass of the bee measured directly after a flight trial, once the polyster thread is removed. The bee is not fed additional nectar and it is not starved beforehand.
2. Starved (empty) body mass: The bee is kept in a petri dish with a wet paper towel, but no nectar, for 24 h. This arrangement allows the bee to move freely, metabolizing nectar without desiccating. Starved body mass is measured after 24 h. Bees were frozen for subsequent measurements.
3. Wing length and IT span: Bees were removed from the freezer and photographed under a dissecting microscope. Bees were manipulated with forces to show the full length of the wings and the top of thorax (to view the wing bases and tegulae). Image J (v1.53f51) was used to measure the length of one haphazardly selected forewing, from the tegulae to the wing tip, and the distance between the two tegulae – i.e., the intertegular (IT) span. Bees were returned to the freezer after the photographs or processed immediately for dry body mass (see step #4).
4. Dry body mass: dry body mass, bees were enclosed in foil packets and dried to a constant mass in a drying oven at 45ºC.
